# Screening of an annexin‐A2‐targeted heptapeptide for pancreatic adenocarcinoma localization

**DOI:** 10.1002/1878-0261.13352

**Published:** 2022-12-30

**Authors:** Heyao Ma, Kai Jiang, Yuhan Hong, Yu Lei, Yue Fan, Wenjian Jiang, Lei Zhao, Jinyang Liu, Weifan Yao, Jiao Xu, Miao He, Minjie Wei

**Affiliations:** ^1^ Department of Pharmacology, School of Pharmacy China Medical University Shenyang China; ^2^ Liaoning Key Laboratory of Molecular Targeted Anti‐Tumor Drug Development and Evaluation China Medical University Shenyang China; ^3^ Liaoning Cancer Immune Peptide Drug Engineering Technology Research Center China Medical University Shenyang China; ^4^ Key Laboratory of Precision Diagnosis and Treatment of Gastrointestinal Tumors, Ministry of Education China Medical University Shenyang China; ^5^ Department of Hepatobiliary and Pancreatic Surgery First Hospital of China Medical University Shenyang China; ^6^ Liaoning Medical Diagnosis and Treatment Center Shenyang China

**Keywords:** *ANXA2*, imaging, near‐infrared fluorescent, pancreatic adenocarcinoma, peptide

## Abstract

Annexin A2 (*ANXA2*) encodes an oncoprotein whose expression has been found to correlate with poorer overall survival (OS) of pancreatic adenocarcinoma (PAAD) patients. Although peptides are available for targeting *ANXA2*, none of these were initially selected to target this protein specifically. Here, we took *ANXA2* as a molecular target for PAAD and employed the phage display technique to screen for a new *ANXA2*‐targeted peptide. The resultant heptapeptide, YW7, was firstly labeled with fluorescein isothiocyanate (FITC) to evaluate its selectivity in cellular uptake, and further with the near‐infrared fluorescent (NIRF) dye Cy7 to assess *in vivo* distribution in a mouse model bearing PANC‐1 human pancreatic cancer xenografic tumors. We found that both FITC‐YW7 and Cy7‐YW7 probes showed significantly higher uptake in PANC‐1 cells compared to the HPDE6‐C7 pancreatic epithelium cells. Mice intravenously injected with Cy7‐YW7 showed higher tumor‐to‐background ratios (TBRs) (~ 2.7‐fold) in tumor tissues compared to those injected with Cy7 alone. Our study suggested that YW7 is a novel peptide targeting *ANXA2* and Cy7‐YW7 is an NIRF probe potentially useful for the early detection of PAAD.

## Graphical abstract

Annexin A2 (*ANXA2*), a promising molecular target, is closely related to the progression and prognosis of malignant PAAD. This study reported a new *ANXA2*‐targeted heptapeptide (YW7) by phage display. Our data demonstrated the prominent tumor selectivity of *ANXA2*‐targeted fluorescent probes.

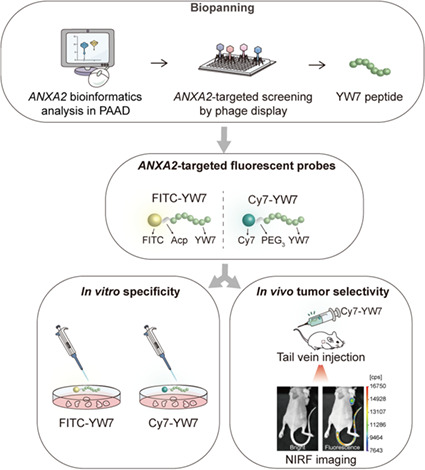

**Abbreviations**
CTX: chlorotoxin
DAB: diaminobenzidine
DFS: disease‐free survival
GTEx: Genotype‐Tissue Expression
H&E: hematoxylin–eosin
HR: hazard ratio
HRP: horseradish peroxidase
IHC: immunohistochemical
IPTG: Isopropyl β‐d‐1‐thiogalactopyranoside
MMP2: matrix metalloproteinase
MOE: Molecular Operating Environment
MRI: magnetic resonance imaging
MST: microscale thermophoresis
NIRF: near‐infrared fluorescent
NPs: nanoparticles
OS: overall survival
PAAD: pancreatic adenocarcinoma
PDC: peptide‐drug conjugates
PDT: photodynamic therapy
PET: positron emission tomography
RP‐HPLC: reverse‐phase high‐performance liquid chromatography
si‐NC: si‐negative control
siRNAs: small interfering RNAs
SPECT: single‐photon emission tomography
TBRs: tumor‐to‐background ratios
TCGA: The Cancer Genome Atlas
TPM: transcripts per million

## 1 Introduction

Pancreatic adenocarcinoma (PAAD) is a malignant digestive tract tumor with an extremely poor prognosis. The 5‐year survival rate of PAAD is only 10% [1]. Thus far, surgery is still the best option for PAAD treatment, but the lack of strategies to diagnose PAAD early unqualifies 80–85% of PAAD patients, who have already developed metastasis at the time of diagnosis, for surgical resection [2]. Furthermore, the inability to locate tumors in preoperative and intraoperative examinations imposes a challenge for complete tumor resection. There is an urgent need for new strategies for early tumor detection [3] and localization [4] to improve the treatment outcomes of PAAD patients. Molecular imaging is a promising tool for non‐invasively characterizing biological events and thus facilitating both preoperative and intraoperative decision‐making [5]. Currently, molecular probes for magnetic resonance imaging (MRI), positron emission tomography (PET), and single‐photon emission tomography (SPECT) have been developed [6] and demonstrated the potential to facilitate early detection of PAAD in preclinical models. For example, a radiolabeled peptide specific to the DNA damage signal, ^111^In‐anti‐γH2AX‐TAT, has been used for the sensitive detection of pancreatic cancer at early stages in mice [7]. In another study, IGF1‐targeted gadolinium‐labeled nanoparticles TBR have been developed as a theranostic platform for both MRI detection and treatment of murine pancreatic cancer models [8]. Therefore, molecular imaging is very important for early detection to achieve timely, accurate, personalized treatment. Based on this, the ability of molecular targeted probes to accurately target tumor biomarkers specific to tumor aggressiveness is of critical importance.

Peptide‐based imaging probes have emerged as an amenable tool to achieve higher tumor permeability and specificity. Many researchers have selected peptides as potential tumor ligands, due to their smaller size and versatility for chemical modification and cost‐effectiveness [9]. Tumor‐targeted peptides can be derived from natural products, bio‐panning libraries, or synthetic peptide arrays. Phage display technology [10] is a classic and efficient way to screen for short peptides against a specific target using the biopanning process. Molecular targeting peptides that screened from phage display, such as VEGFR, EGFR, and HER2‐targeted peptides, have specifically shown high tumor‐specific affinities [11]. Recently, annexin A2, a 36KD calcium‐dependent phospholipid‐binding protein of the annexins family encoded by the *ANXA2* gene, has been identified as a novel biomarker in PAAD as elevated *ANXA2* expression was found to significantly correlate with poorer overall survival (OS) and disease‐free survival (DFS) of PAAD patients [12]. Besides, *ANXA2* has been found to be intracellularly and extracellularly overexpressed in various types of tumors, in which *ANXA2* translocates to the tumor cell membrane and facilitates the generation of plasmin in the extracellular matrix, resulting in enhanced cancer cell invasion and migration [13, 14]. *ANXA2* has been used as a valuable biomarker for both cancer diagnosis and prognosis [15]. Thus far, peptides have been discovered for developing cancer‐specific imaging probes via targeting *ANXA2*. For example, TM601 (a 36‐amino acids peptide) [16], which is a synthetic peptide with the same sequence as chlorotoxin (CTX) derived from the venom of the scorpion Leiurus quinquestriatus, has been found to target both matrix metalloproteinase (MMP2) and phosphorylated *ANXA2* and used to develop an optical imaging probe named BLZ100 for detecting multiple types of tumors. Other CTX‐based bioconjugates, such as ^131^I‐TM601, are also being investigated for tumor detection [17]. In a recent study, a cyclic octapeptide, LS301, was also found to target both integrin receptors and phosphorylated *ANXA2* in the presence of calcium, and the ability of LS301 to emit near‐infrared fluorescence makes it a desirable imaging probe to illuminate tumor margin [18]. However, those peptides were not initially selected to target *ANXA2*. We postulated that peptides that target *ANXA2* would presumably allow selective homing to membrane‐bound *ANXA2* in the PAAD mouse model, because of the low levels of *ANXA2* in non‐tumor tissues. The discovery of such an *ANXA2*‐specific peptide could greatly facilitate further clinical development of diagnostic and therapeutic agents using *ANXA2* as the molecular target.

Using the phage display technique, we identified a heptapeptide YW7 to specifically target the recombinant protein of *ANXA2*. The FITC‐YW7 probe demonstrated a high selectivity in binding PANC‐1 pancreatic cancer cells *in vitro*. We then conjugated YW7 with the NIRF dye Cy7 as the long wavelength of NIRF (700–900 nm) is desirable to achieve higher TBRs in deep tissue, meanwhile reducing autofluorescence [19]. We showed that the molecular fluorescent imaging probe Cy7‐YW7 demonstrated strong PAAD targeting capabilities *in vivo*, making it a promising probe for enhancing the diagnostic efficiency of PAAD.

## 2 Materials and methods

### 2.1 Cell culture and patient tissue samples

A total of four human pancreatic cancer cell lines (BxPC‐3, SW1990, MIA PaCa‐2, and PANC‐1) were purchased from the Cell Bank of the Chinese Academy of Sciences (Shanghai, China). MIA PaCa‐2 and PANC‐1 cells were cultured in DMEM medium supplemented with 100 U·mL^−1^ penicillin, 100 μg·mL^−1^ streptomycin, and 10% FBS (all from Gibco, Carlsbad, CA, USA). Additionally, 2.5% horse serum was added to the medium of MIA PaCa‐2 cells. BxPC‐3 cells were cultured in RPMI‐1640 medium (Gibco) with 100 U∙mL^−1^ penicillin, 100 μg∙mL^−1^ streptomycin, and 10% FBS. SW1990 cells were cultured in L‐15 medium (Gibco) with 100 U∙mL^−1^ penicillin, 100 μg∙mL^−1^ streptomycin, and 10% FBS. The normal human pancreatic ductal cell line HPDE6‐C7 was obtained from Jennio Biotech Co., Ltd (Guangzhou, China) and cultured in RPMI‐1640 medium containing 10% FBS, 100 U∙mL^−1^ penicillin, and 100 μg∙mL^−1^ streptomycin. All cells were grown at 37 °C with 5% CO_2_. Six pairs of human pancreatic cancer tissue samples were obtained from six patients who underwent surgery at the First Affiliated Hospital of Chinese Medical University from 2020. All patients who provided clinical specimens signed the written informed consent form. All the procedures were performed in accordance with the 1964 Declaration of Helsinki principles and its later amendments or comparable ethical standards. All protocols were approved by the Medical Ethics Committee of Chinese Medical University (Approval No. [2020]102).

### 2.2 Peptide synthesis

Solid‐phase synthesis was performed to synthesize peptides and fluorescent dye‐labeled peptides. FITC was labeled to the N‐terminal of YW7 peptide via a spacer of 6‐aminocaproic acid by Sangon Biotech Co., Ltd. (Shanghai, China). Cy7 was linked to the N terminal of YW7 peptide via spacer of PEG_3_ by Chinese Peptide Co., Ltd. (Shanghai, China). YW7 peptide and fluorescent probes were characterized by MALDI‐TOF mass spectrometry and reverse‐phase high‐performance liquid chromatography (RP‐HPLC) and all products had a purity of ≥ 95%.

### 2.3 Bioinformatics analysis

The RNA‐seq data expressed as FPKM and the related clinical information of PAAD were downloaded from The Cancer Genome Atlas (TCGA, http://cancergenome.nih.gov/), and the FPKM data were transformed to transcripts per million (TPM) values for all the samples by using r version 3.6.3 software. Genotype‐Tissue Expression (GTEx) database was obtained from UCSC Xena browser (https://xenabrowser.net/datapages/). Mann–Whitney test was used to detect the differences in expression between tumor and normal tissues. The overall survival (OS) and progression‐free survival (PFS) result was expressed as Kaplan–Meier (K‐M) curve by “survminer” and “survival” r package. Univariate Cox regression analysis was implemented to evaluate prognostic analysis using r version 3.6.3 software. The nomogram was further established to predict cancer prognosis individually. The criterion for significance was *P* < 0.05 for all comparisons.

### 2.4 Animals

The 4‐week‐old male BALB/c nude mice were purchased from Beijing Hfk Bioscience Co., Ltd. (Beijing, China) and raised at the animal facility of China Medical University. Each mouse was subcutaneously injected with 1 × 107 PANC‐1 cells into the right flanks. Mice were housed at 22 ± 1 °C and kept on 12‐h light/dark cycles, with 50% humidity. Distilled water and standard mouse chow diet were provided *ad libitum*. All animal studies were carried out in accordance with the guidelines published by the Institutional Animal Care and Use Committee of China Medical University (Shenyang, China). All protocols were approved by the Medical Ethics Committee of Chinese Medical University (Approval No.CMU2020320).

### 2.5 Western blotting analysis

*ANXA2* expression in human cells and patients' tissues was determined by western blotting. Cells were lysed on ice in RIPA Lysis Buffer (Beyotime, Shanghai, China) with 1% PMSF for 30 min. Fresh tissues were suspended in RIPA Lysis Buffer with 1% PMSF, homogenized for 2 min, and then lysed on ice for another 30 min. The supernatant was collected and the protein concentration was determined after centrifuging for 10 min at 8000 ***g***. The protein concentration was detected by BCA Protein Assay Kit (Beyotime). Proteins extracts were separated by 12% SDS/PAGE gel and then transferred to the PVDF membranes. Next, the membranes were blocked with 5% skimmed milk powder at room temperature for 1 h. Rabbit primary antibodies specific to *ANXA2* (1 : 1000; Cell Signaling Technology, Billerica, CA, USA; #8235S) were used to incubate the membranes at 4 °C overnight. Anti‐β‐tubulin mouse primary antibodies (1 : 5000; EarthOx Life Science, Millbrae, CA, USA; E021040‐01) were applied as a loading control. On the following day, the membranes were incubated with the secondary antibodies conjugated with horseradish peroxidases at room temperature for 1 h. ECL reagents (Millipore, Billerica, CA, USA; WBULS0500) were applied to the membranes in the dark and protein bands were visualized by the chemiluminescence imaging system. The band density was quantified by imagej software (National Institutes of Health, Bethesda, MD, USA).

### 2.6 Recombination and purification of *ANXA2*

*ANXA2* protein expression plasmid was constructed by inserting the coding sequence of GST‐fused *ANXA2* into pGEX‐6P‐1 plasmid by OBiO Technology Corp. Ltd., Shanghai, China. using the BamHI and XhoI enzymic sites. The recombined plasmid, pGEX‐6P‐*ANXA2* or pGEX‐6P‐1, was then transfected into BL21 (DE3) *E. coli*. Isopropyl β‐d‐1‐thiogalactopyranoside (IPTG) was added to the culture medium when OD600 of the bacteria culture reached 0.6 to induce *ANXA2* expression. After induction, and the culture flask kept shaking for another 5 h, the bacteria were collected and lysed with lysozyme. Finally, the resulting supernatant was loaded to GST tag purification resin (Beyotime), and the resin was washed with PBST buffer six times before adding elution buffer and collecting elutions. Purity and product size were evaluated by SDS/PAGE and Coomassie blue staining.

### 2.7 Phage display screening toward *ANXA2*

The Ph.D.‐7 phage display library (New England Biolabs, Beverly, MA, USA) was used to screen candidate peptides with high affinity toward *ANXA2* but not GST. A quantity of 0.5 μg of GST or *ANXA2* recombinant protein was immobilized on a 96‐well plate overnight. 1% BSA was used to block nonspecific binding. In the first round of panning, phage solution was applied to wells coated with GST and incubated for 1 h with gentle shaking. Subsequently, unbound phages were collected by aspirating the remaining solution and then added to GST‐fused *ANXA2* recombinant protein by gentle shaking. The bound phages were eluted with 0.2 m glycine‐HCl buffer (pH 2.2) of 100 μL, followed by neutralizing with Tris–HCl (pH 9.1). The collected phages were titrated on LB‐ampicillin/IPTG/X‐gal plates and amplified by culturing with *E. coli* (ER2758) and the amplified phages were used for another round of panning following the aforementioned procedures. In the third round of panning, eluted phages were cultured on an LB‐ampicillin/IPTG/X‐gal plate and eventually, phages were randomly selected for DNA extraction and sequencing. DNA sequencing services were provided by Sangon Biotech Co., Ltd.

### 2.8 Microscale thermophoresis (MST) assay

The binding affinity of YW7 peptide to *ANXA2* was measured by MST through Monolith NT.115 instrument (NanoTemper Technologies, Munich, Germany). In MST assay, YW7 was labeled with FITC and the concentration was kept unchanged. The unlabeled *ANXA2* protein was diluted according to the gradient. The reaction solution was MST buffer containing 0.05% Tween‐20. After a short time of binding reaction, the sample was loaded into NT.115 standard capillary. The resulting dissociation constant (*K*_D_) and binding curve were calculated by the MO. Control Analysis software (NanoTemper).

### 2.9 Molecular operating environment (MOE) assay

Docking analysis of YW7 to *ANXA2* protein was performed by MOE Version 2020.09 (Chemical Computing Group Inc., Montreal, Canada) according to compute energy scoring functions. The structure of *ANXA2* (PDB:4HRE) was downloaded from the PDB database (https://www.rcsb.org/) and YW7 was generated from the Protein Builder Module of MOE. The spatial structure of YW7 was optimized by the Energy Minimize Function of MOE. Docking was conducted automatically by default parameters. The structure with the lowest free energy of binding was selected.

### 2.10 Evaluating YW7 binding to *ANXA2* using affinity column and LC/MS detection

To prepare the *ANXA2*‐immobilized affinity column, CNBr‐activated Sepharose (GE Healthcare Bio‐Sciences AB, Uppsala, Sweden) was swelled by 1 mm HCl and coupling buffer. Purified recombinant *ANXA2* of 0.7 mg was loaded onto the resin and incubated at 4 °C overnight. After blocking with ethanolamine, the resin was washed with 50 mm Tris–HCl (pH 8.0) and 50 mm Glycine‐0.5 m NaCl (pH 4.0) buffers alternatively for three rounds. Then, the resin was washed with PBS and 1 mg YW7 dissolved in DEPC was added to the *ANXA2*‐loaded resin. After binding for 1 h at 4 °C, the resin was washed again with 50 mm Tris–HCl (pH 8.0) and 50 mm Glycine‐0.5 m NaCl (pH 4.0) buffers. Eluents were eventually mixed with 50% acetonitrile and analyzed by UPLC‐G2XS‐QTOF/MS (Waters, Milford, MA, USA).

### 2.11 Characterizing the binding of FITC‐YW7 to cells

We suspended cells in a concentration gradient of FITC‐YW7 (0–1.5 μm) at 37 °C for 30 min. Then, cells were washed with PBS three times and resuspended in PBS and finally transferred into a black 96‐well plate (1 × 10^5^ per well). Fluorescence intensity of the cells was measured by a fluorescence multimode microplate reader (Tecan, Mnnedorf, Switzerland) at an excitation wavelength 490 nm and emission wavelength 518 nm. The apparent dissociation constant *K*_d_ was calculated by fitting the data to nonlinear binding curves using the embedded function of graphpad prism 7.0 (GraphPad Software, Inc., San Diego, CA, USA).

### 2.12 SiRNA knockdown of *ANXA2*

Small interfering RNA (primer sequences: forward 5′‐AGACCAAAGGUGUGGAUGAUU‐3′ and reverse 5′‐UCAUCCACACCUUUGGUCUUU‐3′) was used to downregulate the expression of *ANXA2* (si‐*ANXA2*). A scrambled sequence siRNA was used as a negative control and a blank group was treated without siRNA. PANC‐1 cells were seeded in a six‐well plate with serum‐free medium and treated with siRNAs and lipofectamine 3000 reagent (Invitrogen, Carlsbad, CA, USA). At 48 h after transfection, cells were harvested, and total RNA and proteins were extracted. Knockdown efficiency was evaluated by western blotting and quantitative real‐time PCR. Primer sequences were constructed by Sangon Biotech Co., Ltd.

### 2.13 Measurement of cellular uptake by flow cytometry and fluorescence imaging

PANC‐1 and HPDE6‐C7 cells were seeded in six‐well plates at a density of 3 × 10^5^ cells·mL^−1^ and incubated overnight. The cells were incubated with 25 μm FITC‐YW7 for 30 min and 1 h for flow cytometry analysis of bound and internalized peptide, and cells were incubated with 1, 2, 4 μm FITC‐YW7 or Cy7‐YW7 for 1 h for fluorescence imaging of the live cells. Cells were washed with PBS three times and centrifuged for 5 min after 37 °C incubation. Si‐*ANXA2‐* or si‐NC‐transfected PANC‐1 cells were detached with nonenzymatic dissociation solution from six‐well plates. 20 μm FITC‐YW7 was added to the suspended cells and incubated at 37 °C for 15 min. Cells were washed with PBS and centrifuged for 5 min repeating three times. The supernatant was then discarded and the cells were resuspended in PBS. Percent of total cells with fluorescent signals was determined by flow cytometry using a FACSCelesta flow cytometer (BD Biosciences, San Jose, CA, USA). Fluorescence intensity was quantified by an imaging system (Berthold LB983, Bad Wildbad, Sttugart, Germany).

### 2.14 Immunofluorescence

Patients' pancreatic tumor tissues were embedded in OCT embedding medium, cut into 4 μm slices and frozen at −80 °C. Cells were seeded in 24‐well plates with glass coverslips and cultured overnight. The following day, frozen tissue sections were fixed with cold acetone, and cells were fixed with 4% paraformaldehyde. FITC‐YW7, anti‐*ANXA2* primary antibody, TRITC‐labeled goat anti‐rabbit IgG (1 : 200; EarthOx Life Science, E031320‐02), and DAPI (Beyotime, C1002) were added to tissues or cells followed by blocking with 3% BSA and goat serum separately. Cells or tissue cells were visualized under a confocal laser scanning microscope (C2 plus, Nikon, Japan) at 408 nm (blue), 488 nm (green), and 543 nm (red).

### 2.15 *In vivo* and *ex vivo* imaging

Mice‐bearing tumors about 6–8 mm in diameter (about 6 weeks after injection) were divided into two groups and Cy7 or Cy7‐YW7 (8 nmol diluted in 100 μL) was injected intravenously into mice, respectively. Fluorescence signals of mice were acquired in a live imaging system (Berthold LB983 Night OWL II) using a red filter set (700–780 nm wavelength). During imaging, mice were anesthetized with 2% isoflurane. For *ex vivo* imaging, mice were sacrificed 3 h after probe injection, and the hearts, livers, spleens, lungs, kidneys, and tumors were collected and then imaged. Tumor tissues were fixed in 4% paraformaldehyde for further histological analysis. Fluorescence signals of different organs were measured by indigo software (Bad Wildbad, Stuttgart, Germany) and the binding specificity was gauged by TBRs.

### 2.16 Hematoxylin–eosin (H&E) and immunohistochemical (IHC) staining

Patients' tissues and tumor tissues from PANC‐1‐derived xenografts were fixed in 4% paraformaldehyde overnight at 4 °C. The fixed tissues were dehydrated, embedded in paraffin, and cut into 4 μm slices. The slices were deparaffinized by heating at 65 °C for 2 h. Heat‐induced epitope retrieval was conducted using citrate buffer at 100 °C for 20 min and 3% H_2_O_2_ was used to remove the endogenous peroxidase. After blocking with goat serum for 30 min, tissue slices were incubated with anti‐*ANXA2* antibody overnight at 4 °C. The next day, the tissue slices were incubated with the secondary antibody conjugated to horseradish peroxidase (HRP) at 37 °C for 60 min and diaminobenzidine (DAB) for 3 min. Finally, hematoxylin and eosin (H&E) staining was done and images were obtained under a microscope (NIKON ECLIPSE E200, Tokyo, Japan).

### 2.17 Statistical analysis

In the present study, statistical analyzes and graphics were evaluated by graphpad prism 7.0 (GraphPad Software, Inc.), spss 19.0 software package (SPSS Inc., Chicago, IL, USA), and r software version 3.6.0. Comparation of the differences between groups was processed by Student's *t*‐test and Mann–Whitney test. Survival analysis was estimated using the Kaplan–Meier method, and any differences in survival were evaluated with the log‐rank test. The hazard ratio (HR) and 95% CI associated with the expressions of h‐prune were estimated through a multivariable Cox regression model to assess the association between *ANXA2* expression and OS or PFS. *P* < 0.05 was considered statistically significant. Quantitative data were expressed as mean ± SD.

## 3 Result

### 3.1 *ANXA2* acts as a potential independent survival and prognostic factor in pancreatic adenocarcinoma

We compared the expression levels of *ANXA2* in 178 PAAD tissues of The Cancer Genome Atlas (TCGA) and 171 normal pancreatic tissues of GTEx database of TCGA; *ANXA2* was expressed at higher levels in PAAD tissues than in normal pancreatic tissues (*P* < 0.0001, Fig. S1A). The survival analysis showed that high levels of *ANXA2* expression were associated with poor OS (*n = 88*, *P* < 0.001) and PFS (*n = 88*, *P* < 0.001) in PAAD (Fig. S1B). We further applied the multivariable Cox regression model to investigate the independent prognostic force of the signature. Multivariable analysis results indicated that *ANXA2* (HR = 1.001, 95% CI = 1.000–1.002; *P* = 0.007) have prognostic value for the OS, *ANXA2* (HR = 1.001, 95% CI = 1.000–1.002; *P* = 0.001), residual tumor (HR = 2.311, 95% CI = 1.565–3.413; *P* < 0.001) and tumor position (HR = 2.613, 95% CI = 1.146–5.956; *P* < 0.022) had prognostic value for the PFS. These results demonstrated that *ANXA2* is an independent prognostic factor in PAAD. Age, T, N, grade, stage, and tumor position and residual tumor are also potential‐independent prognostic indicators of PAAD (Fig. S1C). We further constructed the nomograms based on *ANXA2* and other independent prognosis factors (age, gender, grade, stage, and anatomic neoplasm subdivision), in order to demonstrate *ANXA2* is important to predict the survival probabilities of PAAD patients. The expression of *ANXA2* makes the greatest contribution to the prediction of 1‐, 2‐, and 3‐year OS, and calibration curves demonstrated that this nomogram was close to the actual survival duration indicating the superior predictive capacity (Fig. S1D).

**Fig. 1:**
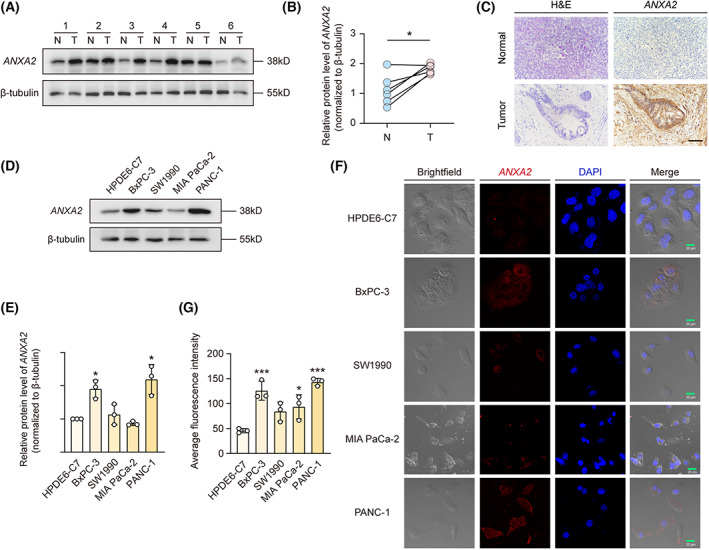
High expression of *ANXA2* in PAAD patient tissues and cells. (A, B) The relative protein level of *ANXA2* was detected by western blotting in PAAD patients' tumor (T) and normal adjacent pancreas (N) tissues (*n = 3* for each tissue). *P* values were determined using paired Student's *t*‐test. (C) Representative image of immunohistochemical staining of *ANXA2* in PAAD patient tissue (tumor) and normal adjacent pancreas (normal) tissue (*n* = 2). Scale bar, 100 μm. (D, E) The relative protein level of *ANXA2* was detected by western blotting in HPDE6‐C7 and indicated PAAD cell lines (*n* = 3 for each cell). Scale bar, 20 μm. *P* values were determined using paired Student's *t*‐test. (F, G) Localization of *ANXA2* using confocal images (red, collected at 543 nm). All the data are represented as mean ± SD. *P < 0.05, ***P < 0.001.

To further investigate *ANXA2* expressions in PAAD, patients' tissues from the First Affiliated Hospital of Chinese Medical University were collected. Proteins in PAAD patients' tissues and PAAD cell lines (BXPC‐3, SW1990, MIAPaCa‐2, PANC‐1) were detected by western blotting. As shown in Fig. 1A–C, PAAD tissues showed a markedly higher expression compared with their counterparts. The highest level of *ANXA2* was observed in PANC‐1 cells and BXPC‐3 cells, and the normal pancreatic cells HPDE6‐C7 exhibited a relatively lower level of *ANXA2* expression (Fig. 1D,E). Localization and expression were confirmed by detecting fluorescent‐labeled *ANXA2* antibody in PAAD cells (Fig. 1F,G). Collectively, these data suggested that abnormally expressed *ANXA2* may be a potential prognostic predictor in the highly malignant PAAD.

### 3.2 Identification of *ANXA2* ‐targeted heptapeptide YW7 by phage display

**Fig. 2:**
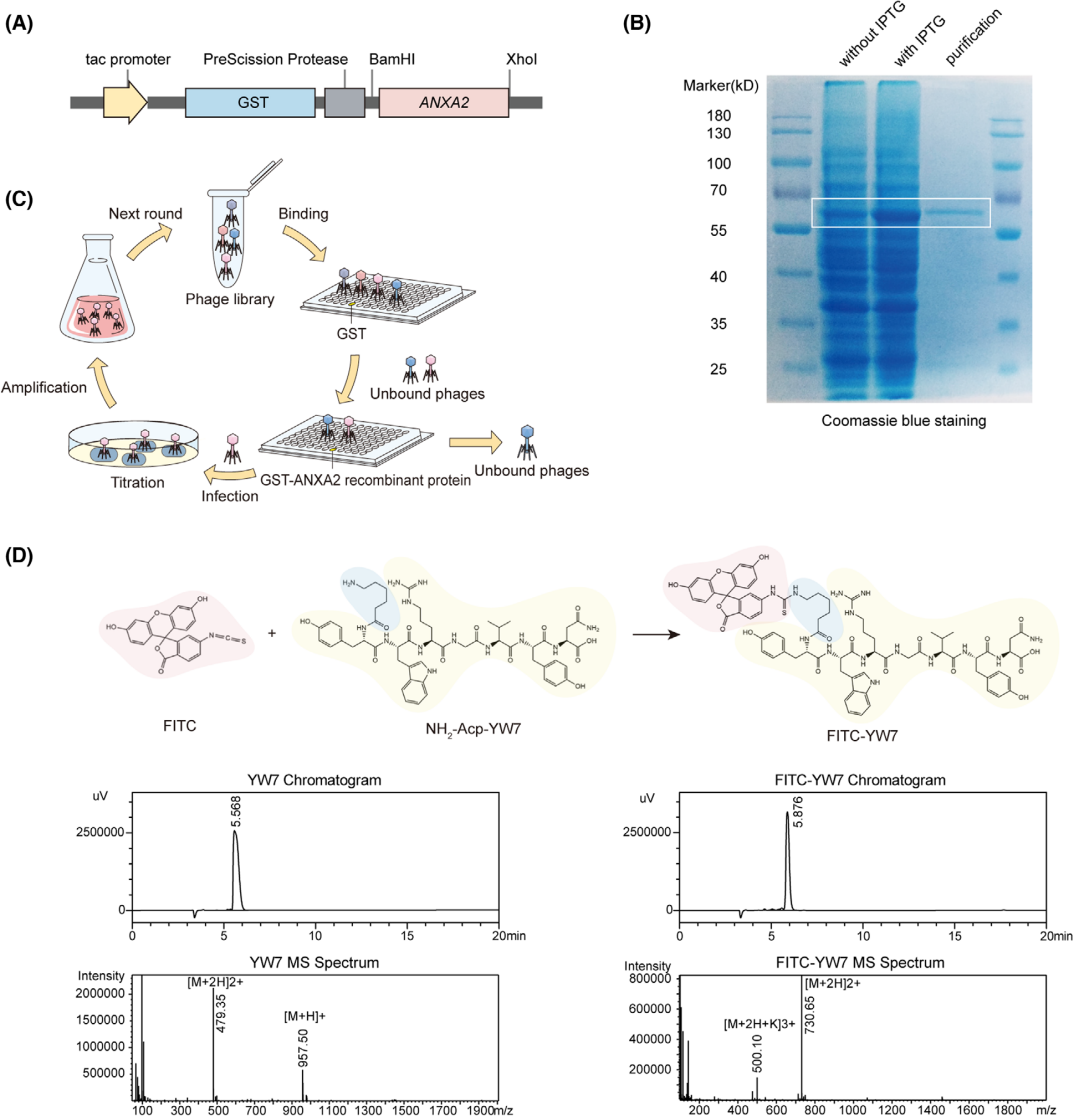
Screening of *ANXA2*‐targeted heptapeptide via phage display. (A) Construction of DNA‐encoding GST‐*ANXA2*‐expressing plasmid. (B) The stained gel of SDS/PAGE with the lanes of BL21 (DE3) cell lysate without IPTG induction, 5.5 h induction with IPTG, and purified GST‐*ANXA2* solution from the lysate (*n* = *2*). (C) Schematic of the phage display for *ANXA2*‐binding heptapeptide. (D) Chemical structure and quality confirmation of the peptides. Purities of YW7, FITC‐YW7 were all above 95% by high‐performance liquid chromatography. Molecular weights were verified by mass spectrometry.

To screen for an *ANXA2*‐targeted peptide, we first established a GST‐*ANXA2*‐inserted *E. coli* vector (pGEX‐6P‐1) as shown in Fig. 2A. The vector was transformed into BL21(DE3) bacteria, followed by IPTG induction at 22 °C. We purified the GST‐*ANXA2* and GST protein using GST‐tag affinity purification, and 12% SDS/PAGE was performed to confirm the size (62 kD) and purity of the products (Fig. 2B). Then, phage display was carried out following steps in the flow chart as shown in Fig. 2C. In phage display, we used GST‐*ANXA2* as the target protein and GST as a negative binding protein, on which high‐throughput screening of an M13 phage display library was performed. We achieved a high enrichment of *ANXA2*‐binding phages after three rounds of screening (Fig. S2A). After DNA sequencing and amino acid translation, we obtained a peptide with the sequence of YWRGVYN among three randomly sequenced phage colonies, and we named the peptide YW7 (Fig. S2B). Peptide YW7 and FITC‐labeled YW7 (purity > 95%) were synthesized by solid‐phase synthesis for further confirmatory experiments (Fig. 2D).

### 3.3 Confirmation of the binding affinity of YW7 to *ANXA2*

**Fig. 3:**
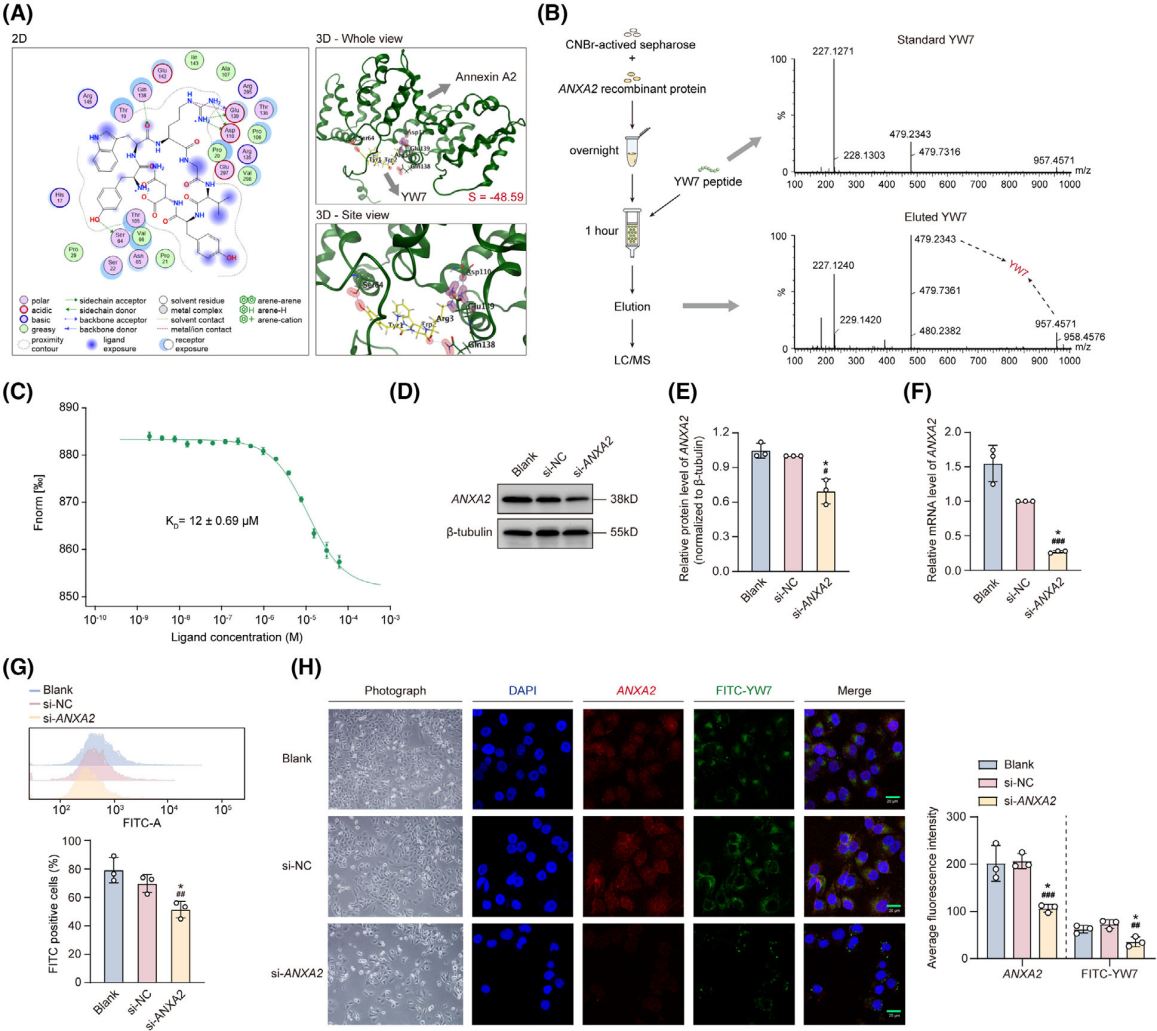
Validation of targeting specificity of FITC‐YW7 to *ANXA2*. (A) Two‐dimensional (2D) and 3D schematic of *ANXA2* (green, PDB:4HRE) docking with structure optimized peptide YW7 (yellow) by MOE. (B) YW7 peptide was eluted from *ANXA2*‐loading column after incubation with *ANXA2* recombinant protein for 1 h and identified. (C) MST assay and *K*_D_ values of enriched FITC‐YW7 toward GST‐*ANXA2* (repeated twice). (D–F) Downregulated *ANXA*2 relative protein (D, E) and mRNA (F) levels after transfected with si*ANXA2* in PANC‐1 cells for 48 h (*n = 3*). (G) Identification of the binding efficiency of FITC‐YW7 to si‐*ANXA2* compared to – si*ANXA2* or si‐NC transfected PANC‐1 cells by flow cytometry (*n = 3*). (H, I) The fluorescent images of FITC‐YW7 toward PANC‐1 cells transfected with si‐*ANXA2*, si‐NC, or blank (*n = 3*). Scale bar, 20 μm. Data are represented as mean ± SD. *P* values were determined using paired Student's *t*‐test. *P < 0.05, **P < 0.01, ***P < 0.001 versus blank; ^##^ *P* < 0.01, ^###^ *P* < 0.001 versus si‐NC.

To confirm *ANXA2* is the binding target of YW7, MOE was utilized to perform preliminary characterization of the affinity of YW7 toward *ANXA2*. The structure of *ANXA2* (PDB:4HRE) was downloaded from PDB database, and the structure of YW7 was optimized by the MOE‐protein builder module. After the molecular docking, the binding mode of *ANXA2* in complex with YW7 peptide with the lowest free energy was selected and further analyzed as shown in Fig. 3A. It can be seen that Ser64 and Gln138 of *ANXA2* formed H‐bond with Tyr1 and Trp2 residues of YW7; in addition, both Asp110 and Glu139 of *ANXA2* formed an ionic bond and a H‐bond with the Arg3 residues of YW7. These interactions proved that YW7 has a good affinity (*SVM classifier* = −48.59) to *ANXA2*. To further confirm the binding affinity of YW7, resin fixed with *ANXA2* overnight was loaded to a column. Then, YW7 was added to the column and eluted after 1 h binding from the column and LC/MS was utilized for the characterization of bound YW7 (Fig. 3B). We also tested the binding affinity between FITC‐YW7 and *ANXA2* by MST. MST data suggested FITC‐YW7 binds to *ANXA2* with a dissociation constant (*K*_D_, app) of 12 ± 0.69 μm (Fig. 3C). To further verify that the binding between YW7 and *ANXA2* on tumor cells, we further knocked down of *ANXA2* on PANC‐1 cells using small interfering RNAs (siRNAs). Downregulation of *ANXA2* after knockdown was verified by both western blotting and quantitative real‐time PCR analysis after knockdown (Fig. 3D–F). The fluorescence signal of FITC‐YW7 was detected by flow cytometry and fluorescent microscopy after binding with PANC‐1 cells. As expected, we found that PANC‐1 cells with lower expression of *ANXA2* were demonstrated a lower fluorescence signal intensity toward FITC‐YW7 (Fig. 3G–I) and to verify that *ANXA2* overexpression is essential for the binding of FITC‐YW7 on PANC‐1 cells.

### 3.4 Selectivity of FITC‐YW7 on PANC‐1 cells *in vitro*

**Fig. 4:**
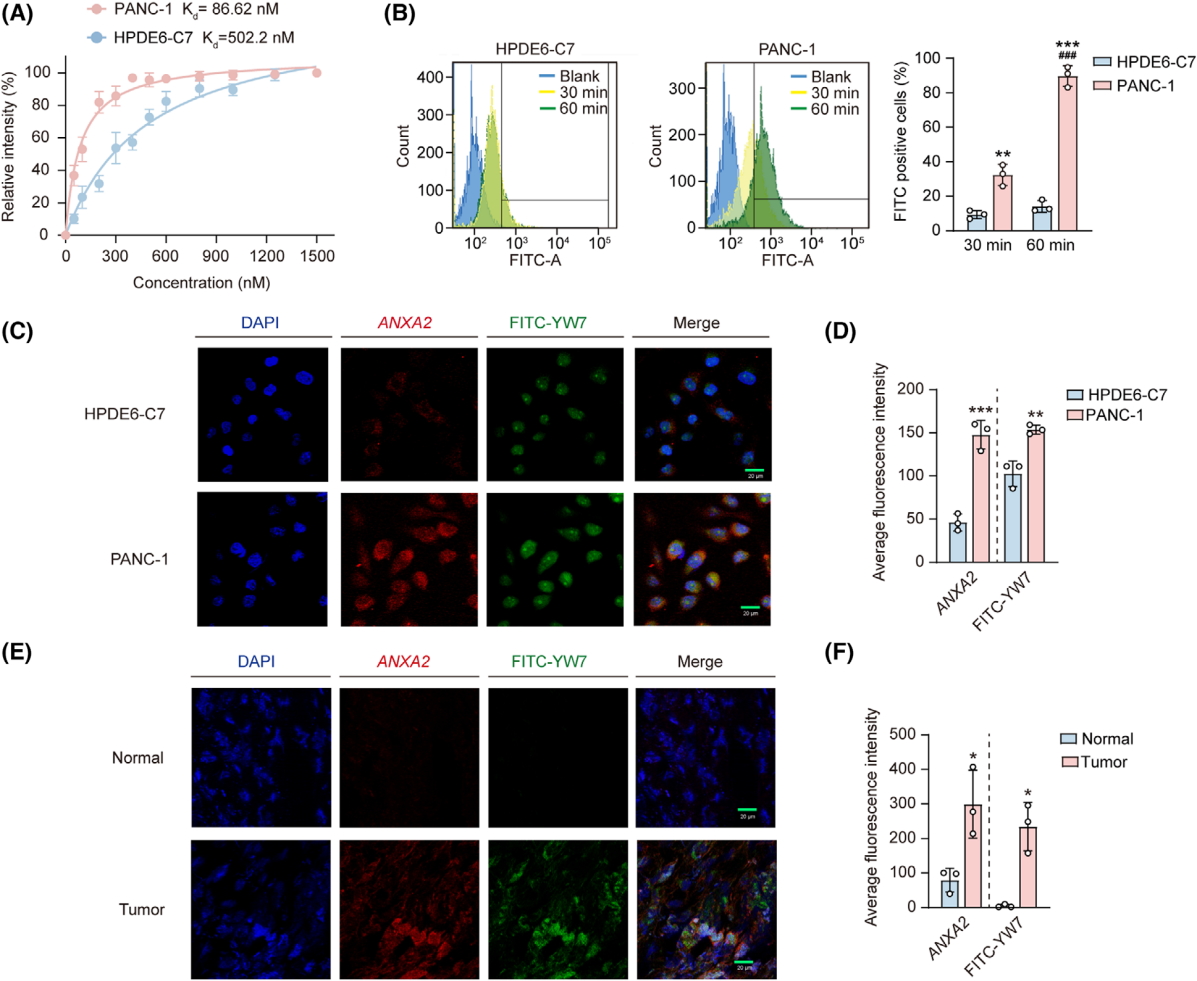
Selective binding of FITC‐YW7 toward PAAD cancer cells and tissues. (A) Binding affinity of the FITC‐YW7 with PANC‐1 and HPDE6‐C7 cells. (B) Internalization of FITC‐YW7 toward HPDE6‐C7 and PANC‐1 cells after 30 min and 1 h binding time at 37 °C. (C–F) Representative fluorescent images of the colocalization of FITC‐YW7 (green, collected at 488 nm) and *ANXA2* (red, collected at 543 nm) in HPDE6‐C7 and PANC‐1 cells (C, D) and PAAD patients' tumors and normal adjacent pancreas tissue frozen sections (4 μm slice) (E, F) by fluorescence detection. Scale bar: 20 μm. The intensity of red and green fluorescence was shown in the bar graph. All the experiments were repeated three times. Data are represented as mean ± SD. *P* values were determined using paired Student's *t*‐test. *P < 0.05 **P < 0.01, ***P < 0.001 versus HPDE6‐C7; ^###^ *P* < 0.001 versus PANC‐1 30 min.

In the above analysis of YW7, *ANXA2* was verified to be the binding target of YW7 and to mediate the binding ability to PANC‐1 cells. Thus, we further explored the binding selectivity of YW7, based on the abnormal expression of *ANXA2* in tumor cells. We characterized the binding of FITC‐YW7 to PANC‐1 cells as shown in Fig. 4A. An apparent binding constant (*K*_d_) of 86.62 nm was obtained for PANC‐1 cells, but a lower *K*_d_ of 502.2 nm for HPDE6‐C7 cells. Next, the cellular uptake of FITC‐YW7 was assessed by flow cytometry. After incubation with FITC‐YW7 (25 μm) for 30 min and 1 h, approximately 90% of PANC‐1 cells demonstrated a strong FITC signal, while only about 10% of HPDE6‐C7 cells showed a strong FITC signal. Moreover, FITC‐YW7 uptake in PANC‐1 cells was shown to be in a time‐dependent manner (Fig. 4B). Thereafter, we incubated PANC‐1 cells with FITC‐YW7 and anti‐*ANXA2* antibodies for confocal microscopy detection. Our data demonstrated that PANC‐1 cells exhibited significantly stronger signals from FITC‐YW7 and anti‐*ANXA2* antibodies, compared to HPDE6‐C7 cells. Moreover, FITC‐YW7 colocalized with anti‐*ANXA2* antibodies on PANC‐1 cells (Fig. 4C,D). Similarly, FITC‐YW7 showed greater binding on *ANXA2* high expressed PAAD tumor than paired normal adjacent pancreas tissues (Fig. 4E,F).

### 3.5 Specifically constructed Cy7‐YW7 probe targeting PANC‐1 *in vitro* and *in vivo*

**Fig. 5:**
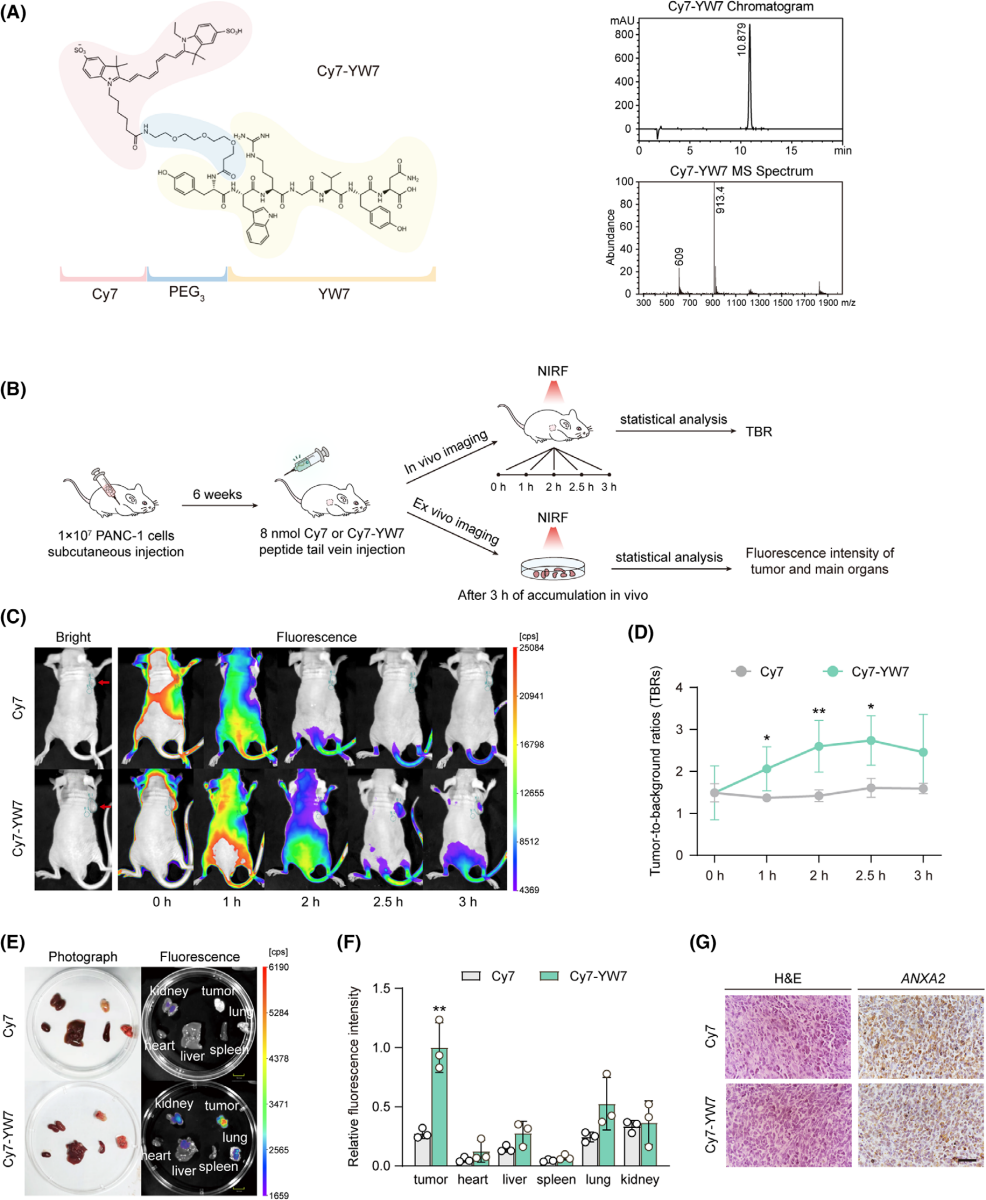
NIRF imaging of Cy7‐YW7 *in vitro* and in PANC‐1 xenograft tumor‐bearing nude mice. (A) Chemical structure of Cy7‐YW7 probe. (B) Schematic diagram of constructing PANC‐1 xenografts in BALB/c nude mice and NIFR imaging procedure. (C, D) *In vivo* distribution and enrichment of Cy7 and Cy7‐YW7. The tumor‐to‐background ratios (TBRs) of Cy7‐YW7 (*n = 4*) were higher than Cy7 (*n = 4*). (E, F) *Ex vivo* fluorescence imaging and relative intensity of tumor xenograft and main organs (*n* = *3*). Scale bar: 10 mm. Quantitative analysis based on relative average fluorescence intensity per organ. (G) Harvested tumor sections (4 μm slice) from PANC‐1 xenograft tumor‐bearing mice for hematoxylin and eosin (H&E) and *ANXA2* antibody staining. Scale bar: 50 μm. Data are represented as mean ± SD. *P* values were determined using an unpaired Student's *t*‐test. *P < 0.05, **P < 0.01.

In our study, we also constructed the Cy7‐YW7 to assess the binding selectivity *in vivo*. First, constructing an NIRF probe target to *ANXA2*, Cy7 fluorescent dye was conjugated to the N terminal of YW7 using a short PEG_3_ linker in between (Fig. 5A). The binding of FITC‐YW7 or Cy7‐YW7 to PANC‐1 cells was observed using a fluorescent imaging system. We found that Cy7‐YW7 (1, 2, 4 μm) were preferred to be internalized by PANC‐1 cells in a dose‐dependent manner. As a control, PANC‐1 cells treated with Cy7 showed a significantly lower fluorescence signal compared to those treated with Cy7‐YW7. However, in the FITC‐YW7 binding assay, PANC‐1 presented lower sensitivity compared to Cy7‐YW7 (Fig. S3A,B). Cy7‐YW7 diluted in PBS at final concentration of 4 μm was used to incubate with PANC‐1 and HPDE6‐C7. Our findings revealed that a stronger fluorescence signal of Cy7‐YW7 was detected after internalization into PANC‐1 cells compared to HPDE6‐C7. Cy7 (4 μm) treated cells have little change in the fluorescence signal (Fig. S3C,D). The *in vivo* pancreatic tumor targeting specificity of Cy7‐YW7 was assessed on mice‐bearing PANC‐1 cell xenografts. Mice with tumors of 6–10 mm in diameter were intravenously injected Cy7‐YW7 or Cy7 (8 nmol, *n = 4* for each group), and images were acquired at 0, 1, 2, 2.5, 3 h after injection (Fig. 5B). Using those injected with Cy7 as controls, our data showed that stronger fluorescence signals in tumor tissues were observed in mice injected with Cy7‐YW7 (Fig. 5C). The highest TBRs of mice injected with Cy7‐YW7 occurred at 2.5 h after injection (TBR_Cy7‐YW7_ = 2.7 ± 0.6, vs. TBR_Cy7_ = 1.5 ± 0.2, *P* < 0.05), and we examined the *ex vivo* tissue distribution of Cy7‐YW7 at 2.5 h after injection (Fig. 5D). As shown in Fig. 5G, substantial retention of Cy7‐YW7 was detected on tumor xenograft, a small amount of fluorescence signal on lung, kidney, liver, and little was on other tissues. Cy7 showed no significant difference in *ex vivo* distribution in major organs except that its tumor tissue accumulation was lower (Fig. 5E,F). *ANXA2* immunostaining also suggested that *ANXA2* is highly expressed in PANC‐1 pancreatic tumor tissue (Fig. 5G).

## 4 Discussion

With the capability of noninvasively recognizing tumor biomarkers with high specificity, molecular imaging is a promising approach to improve the accuracy of tumor detection [20]. Early detection of PAAD is still a clinical challenge due to the lack of imaging biomarkers and the highly fibrotic and dense tumor stroma, which limits its accessibility by imaging agents [21]. Peptides, which are a class of therapeutic and targeting tumor ligands commonly used for constructing molecular imaging probes, have a high biocompatibility and cost‐effectiveness, with their smaller size highly amenable for deep tissue penetration [22]. In this study, we employed the phage display technique to screen for a new heptapeptide specific to *ANXA2*, an oncoprotein overexpressed on the surface of tumor cells during tumor development, and consequently constructing a small‐molecular NIRF probe with a high affinity and selectivity to pancreatic cancer. The choice of *ANXA2* as a biomarker was based on previous reports that high *ANXA2* levels are strongly correlated to the survival of pancreatic cancer patients, which was also verified by our bioinformatical study [12, 23]. We showed that the heptapeptide YW7 has a high binding affinity toward *ANXA2* (*K*_D_ = 12 ± 0.69 μm). Based on YW7, we further developed an *ANXA2*‐specific NIRF probe, Cy7‐YW7, for PAAD detection. In our proof‐of‐concept study, injecting 8 nmol Cy7‐YW7 resulted in rapid tumor accumulation in the mouse‐bearing PANC‐1 tumor xenografts, that is, significant tumor‐to‐background contrast could be seen as early as 1 h after injection and the highest TBRs were observed after 2.5 h. In contrast, Cy7 alone failed to induce enhanced tumor contrast. Thus, Cy7‐YW7, a stable and sensitive NIRF probe, may be further recognized in clinical applications for the imaging of PAAD [24].

To our best knowledge, Cy7‐YW7 is the first *ANXA2*‐specific optical imaging probe that was discovered by phage display. Phage display technology is a traditional biopanning process but the most widely used method to biologically screen specific peptides. Therefore, the screening of peptides by phage display technology mimics the immune selection process by repeating and amplifying of specific binding phages [25]. Despite that phosphorylated *ANXA2* expression is more specific to tumor compared to the unphosphorylated form, our findings show that both FITC‐ and Cy7‐YW7 also possess a high specificity to PANC‐1 cells. Importantly, Cy7‐YW7 was proven to accumulate in tumor with a low background signal (TBRs ~ 2.7). These data support our hypothesis that *ANXA2*‐targeted peptides would selectively target tumor cells as *ANXA2* is commonly overexpressed on tumor cell membranes. Compared to previously identified *ANXA2*‐targeted peptide probes, BLZ‐100 [9], which is based on a 36‐amino acid peptide, and LS301, is based on an octapeptide [18], YW7 has a shorter amino acid sequence of only seven amino acids. Although YW7 and others share the same target of *ANXA2*, the fast clearance and high penetration of Cy7‐YW7 due to the small size are likely other contributing factors of the high TBRs. In addition, as demonstrated in our study and previous studies, *ANXA2* is also abundantly expressed in other types of cancers, making Cy7‐YW7 a potentially valuable probe to detect other *ANXA2*‐positive cancers.

Due to the delayed diagnosis of pancreatic cancer, surgery is still the most effective treatment for pancreatic cancer. However, the recurrence rate of PAAD still remains high (~ 80%) [2]. Our study paves the way for utilizing Cy7‐YW7 to facilitate PAAD localization and delineating margin pre‐, intra‐, or postoperatively. Indeed, the most recently reported *ANAX2*‐targeted LS301 was shown to be capable of illuminating tumor edges due to its selective binding to the invasive front [18]. We expect that Cy7‐YW7 has the potential to address the need to facilitate complete PAAD tumor resection by targeting *ANXA2*‐overexpressing tumor cells. Furthermore, our data also supported the YW7 internalization that followed binding to *ANXA2* in pancreatic cancer cells, suggesting that YW7 has the potential to guide drugs to recognize tumor cells or even cross the cell membrane. Compared with antibody counterparts, YW7 is of smaller size for more efficient tumor penetration. Given that endocytotic and non‐endocytotic pathways are the main mechanism that peptides use to be internalized into cells, YW7 is likely to conduct natural ligand–receptor interactions and induce internalization through endocytosis. However, further data are needed to support this hypothesis. Meanwhile, the YW7 peptide could also serve as a valuable building block for synthesizing new MRI and PET probes, and enable early noninvasive detection of PAAD, which has not been feasible [26, 27]. YW7 may also benefit the construction of novel PAAD‐targeted theranostic agents, such as targeted nanoparticles (NPs), peptide‐drug conjugates (PDC), targeted photodynamic agents for photodynamic therapy (PDT), where specific detection of PAAD through targeting *ANXA2* would greatly enhance their translational potential.

## 5 Conclusions

Annexin A2 (*ANXA2*) is a cancer biomarker and is involved in the occurrence and development of a variety of tumors, particularly PAAD. We have screened an *ANXA2*‐targeting heptapeptide, termed YW7, based on the phage display technique and found that FITC‐YW7 selectively binds to pancreatic cancer cell PANC‐1. Furthermore, we developed a novel NIRF probe Cy7‐YW7. Compared to Cy7‐treated group, Cy7‐YW7 showed enhanced tumor sensitivity and specificity from 1 h after injection in mice‐bearing PANC‐1 cell xenografts. Our data suggested that YW7 is a new *ANXA2*‐ and potential PAAD‐specific peptide. Cy7‐YW7 is a novel NIRF probe that can facilitate PAAD tumor detection by specifically accumulating in tumor tissues *in vivo*. Cy7‐YW7 is a promising molecular imaging probe for early detection and image‐guided surgical resection for PAAD.

## Conflict of interest

The authors declare no conflict of interest.

## Author contributions

HM and MW conceived this study. HM, YF, and YL designed and performed the experiments. YH, HM, and KJ analyzed the data and developed statistical analysis, and drafted the manuscript. JX, WJ, HM, WY, LZ, and JL contributed technical/reagents materials, and/or grant support. HM, LZ, and JL reviewed and edited the document. MH and MW supervised the research. All authors participated in revising the manuscript and agreed to the final version.

### Peer review

The peer review history for this article is available at https://publons.com/publon/10.1002/1878‐0261.13352.

## Supporting information


**Fig. S1.** Survival prognostic value of *ANXA2* in pancreatic cancer. (A) The differential expression level of *ANXA2* expression between tumor and normal tissues on TCGA and GTEx cohorts, which were estimated by the Mann–Whitney test (*P* < 0.001). (B) Kaplan–Meier survival curves showed that patients with lower *ANXA2* expression (*n = 88*) had longer OS (*P* < 0.001) and PFS (*P* < 0.001) than those with higher expression of *ANXA2* (*n = 88*). *P* values were determined using a log‐rank test. (C) Multivariate Cox regression of *ANXA2* as a prognosis factor for OS and PFS of PAAD patients in TCGA. (D) Construction of a prognostic nomogram of PAAD patients by integrating the *ANXA2* expression and other independent prognostic indicators (age, gender, grade, stage, and anatomic neoplasm subdivision). Calibration curves estimated the deviation of predicated 1‐, 2‐, and 3‐year OS and actual survival duration.

**Fig. S2.** Enrichment and sequencing of *ANXA2*‐binding phages. (A) Progressive enrichment of *ANXA2*‐binding phage clones based on titration. (B) DNA sequencing and amino acid translation result of *ANXA2*‐binding phages.

**Fig. S3.** Fluorescent‐labeled YW7 probes imaging *in vitro*. (A, B) Cell imaging after incubation with fluorescent‐labeled YW7 using a live imaging system by a green filter set (490–525 nm) for FITC‐YW7 or a red filter set (700–780 nm) for Cy7‐YW7. PANC‐1 cells were incubated with the same concentration gradient (1, 2, 4 μM) of either FITC‐YW7 or Cy7‐YW7, *P < 0.05 versus PBS; ^#^ *P* < 0.05 versus Cy7; (C, D) PANC‐1 and HPDE6‐C7 after incubation with Cy7‐YW7: 1: HPDE6‐C7 (PBS); 2: PANC‐1 (PBS); 3: HPDE6‐C7 (Cy7); 4: PANC‐1 (Cy7); 5: HPDE6‐C7 (Cy7‐YW7); 6: PANC‐1 (Cy7‐YW7). Experiments were repeated three times. *P* values were determined using paired Student's t‐test. *P < 0.05 versus HPDE6‐C7 (Cy7‐YW7); ^#^ *P* < 0.05, ^##^ *P* < 0.01 versus PBS; ^$^ *P* < 0.05, ^$$^ *P* < 0.01 versus Cy7.

## Data Availability

The datasets used and/or analyzed during the current study are available from the corresponding author upon reasonable request.
