# Supplementary figures and images for: Screening of an annexin‐A2‐targeted heptapeptide for pancreatic adenocarcinoma localization
Source: Mol Oncol. 2022 Dec 30;17(5):872–86. doi: 10.1002/1878-0261.13352 (PMC10158761; doi:10.1002/1878-0261.13352)

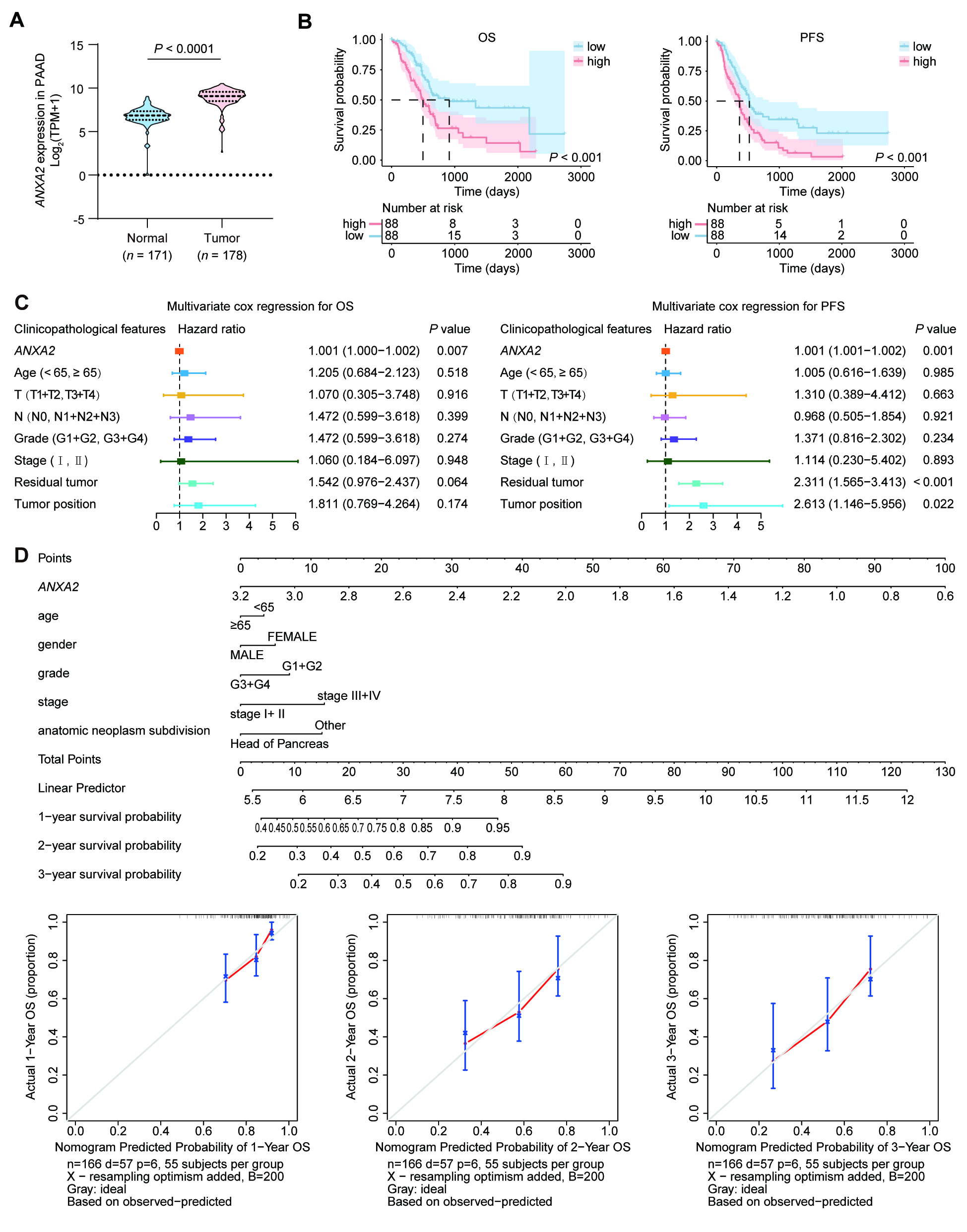

Supplement: Supplementary file 1 — Fig. S1. Survival prognostic value of ANXA2 in pancreatic cancer. (A) The differential expression level of ANXA2 expression between tumor and normal tissues on TCGA and GTEx cohorts, which were estimated by the Mann–Whitney test (P < 0.001). (B) Kaplan–Meier survival curves showed that patients with lower ANXA2 expression (n = 88) had longer OS (P < 0.001) and PFS (P < 0.001) than those with higher expression of ANXA2 (n = 88). P values were determined using a log‐rank test. (C) Multivariate Cox regression of ANXA2 as a prognosis factor for OS and PFS of PAAD patients in TCGA. (D) Construction of a prognostic nomogram of PAAD patients by integrating the ANXA2 expression and other independent prognostic indicators (age, gender, grade, stage, and anatomic neoplasm subdivision). Calibration curves estimated the deviation of predicated 1‐, 2‐, and 3‐year OS and actual survival duration. [file MOL2-17-872-s003.tif]

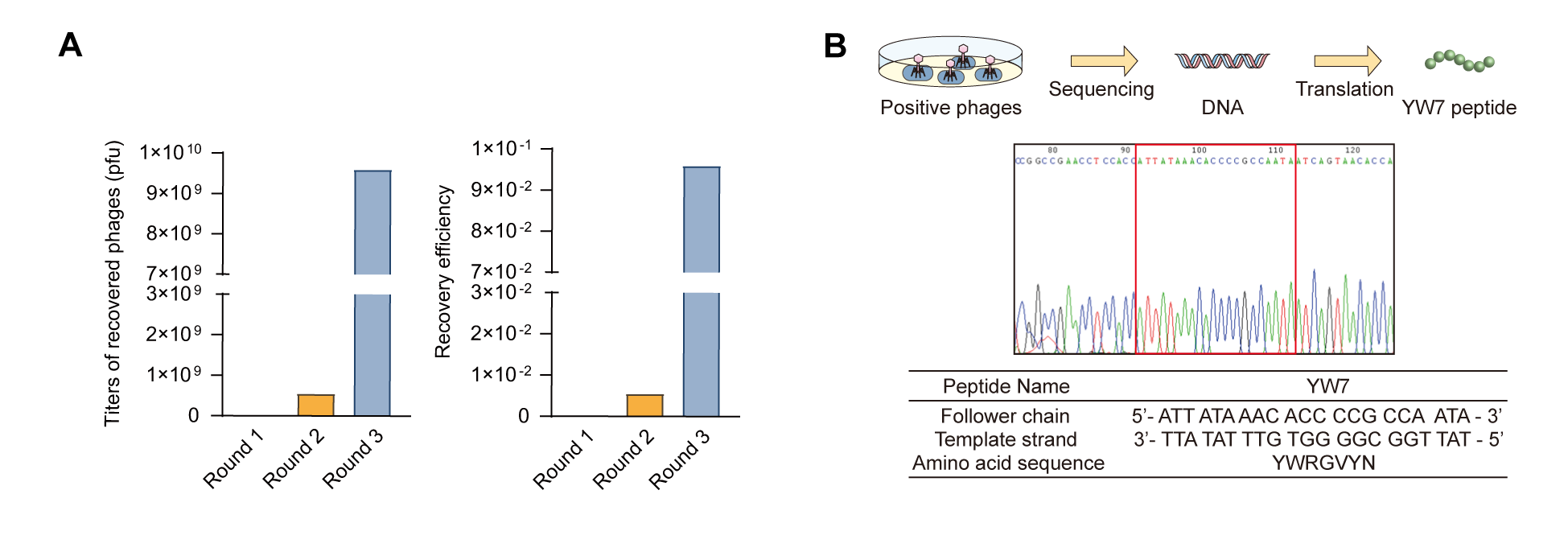

Supplement: Supplementary file 2 — Fig. S2. Enrichment and sequencing of ANXA2‐binding phages. (A) Progressive enrichment of ANXA2‐binding phage clones based on titration. (B) DNA sequencing and amino acid translation result of ANXA2‐binding phages. [file MOL2-17-872-s001.tif]

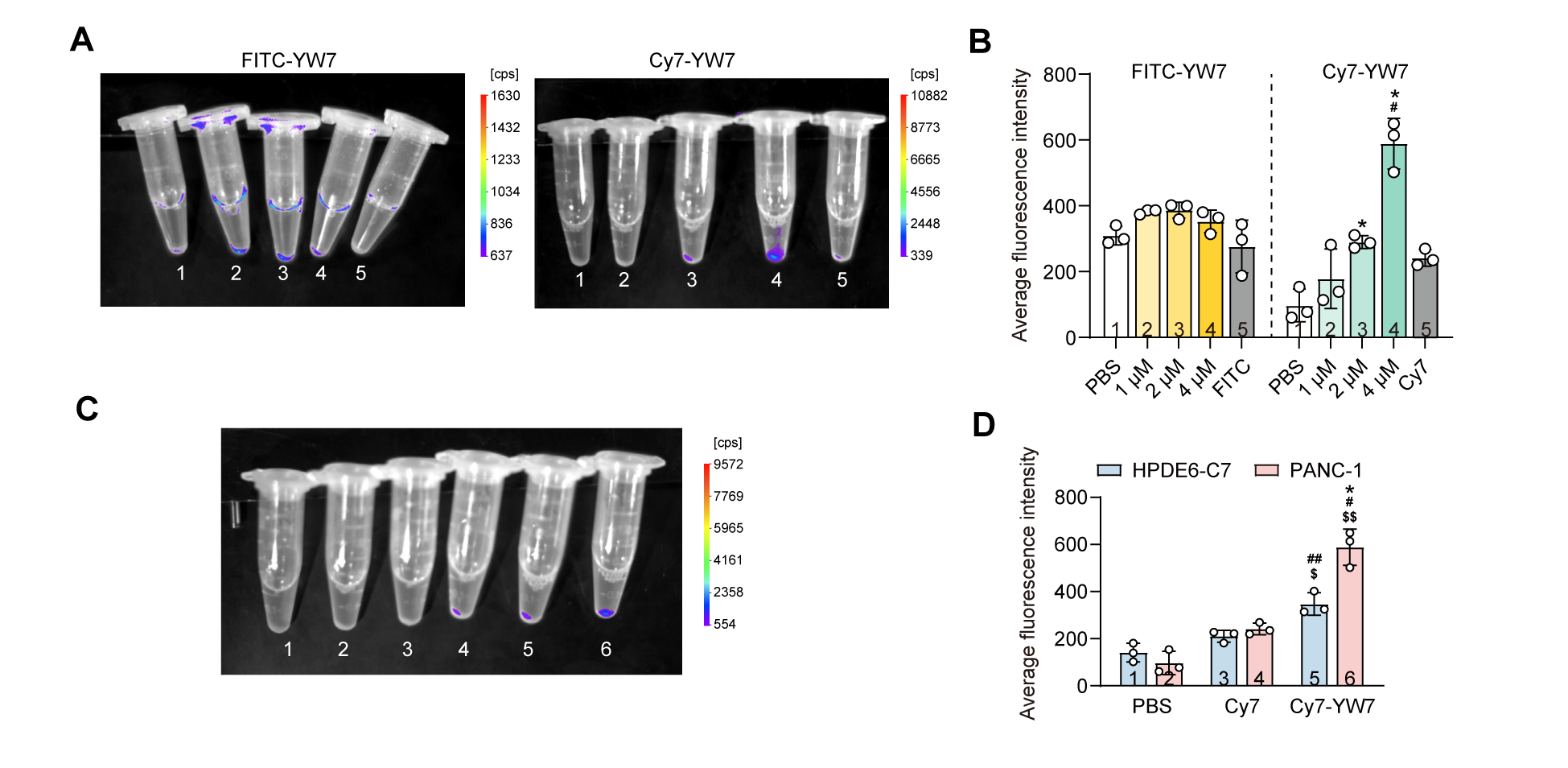

Supplement: Supplementary file 3 — Fig. S3. Fluorescent‐labeled YW7 probes imaging in vitro. (A, B) Cell imaging after incubation with fluorescent‐labeled YW7 using a live imaging system by a green filter set (490–525 nm) for FITC‐YW7 or a red filter set (700–780 nm) for Cy7‐YW7. PANC‐1 cells were incubated with the same concentration gradient (1, 2, 4 μM) of either FITC‐YW7 or Cy7‐YW7, *P < 0.05 versus PBS; # P < 0.05 versus Cy7; (C, D) PANC‐1 and HPDE6‐C7 after incubation with Cy7‐YW7: 1: HPDE6‐C7 (PBS); 2: PANC‐1 (PBS); 3: HPDE6‐C7 (Cy7); 4: PANC‐1 (Cy7); 5: HPDE6‐C7 (Cy7‐YW7); 6: PANC‐1 (Cy7‐YW7). Experiments were repeated three times. P values were determined using paired Student's t‐test. *P < 0.05 versus HPDE6‐C7 (Cy7‐YW7); # P < 0.05, ## P < 0.01 versus PBS; $ P < 0.05, $$ P < 0.01 versus Cy7. [file MOL2-17-872-s002.tif]
